# An extended and validated dataset of game development problems based on postmortem analysis

**DOI:** 10.1016/j.dib.2026.113355

**Published:** 2026-10-14

**Authors:** Claude Cardinal, Bruno Bouchard

**Affiliations:** Université du Québec à Chicoutimi (UQAC), 555, boulevard de l’Université, Chicoutimi, G7H 2B1, Québec, Canada

**Keywords:** Video games, Software engineering, AI-assisted coding, Human validation, Game development

## Abstract

This article presents an extended dataset, along with validation records and quality-control procedures, of software engineering problems reported in video game development postmortems published between 1998 and 2025. The dataset combines the Politowski et al. reference corpus with a contemporary extension collected from Game Developer, Reddit, and the GDC Vault. The extension preserves the original taxonomy and coding structure used in the reference corpus. Problem instances were coded using the original taxonomy and validated through a human-in-the-loop review process supported by AI-assisted extraction. The resulting resource contains 267 postmortems and 1227 coded problem instances, together with source information, temporal metadata, and validation records. The dataset includes source metadata, taxonomy labels, supporting quotations, and validation records that may be reused for software engineering and game development research.

All data records, documentation, and coding procedures are provided to facilitate reuse, reproducibility, and future dataset extensions. The complete dataset, source documents, coding records, validation materials, and associated documentation are available through Zenodo (DOI: 10.5281/zenodo.19683430).

Specifications Table

**Table:** 

Subject	Computer Sciences
Specific subject area	Software engineering, game development postmortem analysis, empirical dataset curation and validation.
Type of data	Text; Spreadsheet; Metadata records; Raw, processed, analyzed, and validated data provided in XLSX, PDF, and TXT formats.
Data collection	Game development postmortems published between 1998 and 2025 were collected from Game Developer (formerly Gamasutra), Reddit, and GDC Vault. Documents were screened using predefined inclusion criteria requiring explicit discussion of “what went right and what went wrong” during development. Candidate problem instances were extracted using Microsoft Copilot and subsequently reviewed through human validation and expert consensus using the taxonomy proposed by Politowski et al.
Data source location	Data was collected from publicly accessible online sources (Game Developer, Reddit, and GDC Vault). The released dataset is maintained by the Department of Computer Science and Mathematics, Université du Québec à Chicoutimi (UQAC), 555 boulevard de l’Université, Chicoutimi, Québec G7H 2B1, Canada.
Data accessibility	Repository name: Zenodo Data identification number: 10.5281/zenodo.19683430 Direct URL to data: https://doi.org/10.5281/zenodo.19683430 The repository contains derived research data and copies of publicly accessible source materials used in this paper. The CC BY 4.0 license applies only to the original research data, metadata, annotations, coding records, and documentation created by the authors. Copyright and licensing conditions governing the original source materials remain with their respective rights holders and are unaffected by the license applied to the repository.
Related research article	None.

## 1 Value of the Data

The dataset contains observations originating from multiple postmortem sources with preserved source provenance, allowing users to conduct source-stratified analyses.

Researchers can reuse this dataset (1227 problem instances from 267 postmortems) for empirical studies in software engineering, game development, or taxonomy validation, with full traceability via source metadata and quotations.

• The dataset preserves the original taxonomy proposed by Politowski et al. while extending coverage with 300 validated problem instances collected between 2019 and 2025, allowing compatibility with previous datasets and replication materials.
• Source provenance is retained for all observations through explicit identification of Game Developer, Reddit, and GDC Vault records, enabling source-aware analyses and dataset filtering.
• The released materials include structured datasets, source documents, coding records, validation documentation, and supporting quotations, facilitating independent verification and reuse of the coding process.
• The accompanying AI-assisted and human-validated coding documentation may be reused as an example dataset curation workflow for future qualitative annotation and dataset maintenance projects.

The released materials provide a documented corpus of coded postmortem observations together with validation records and supporting documentation.

## 2 Background

Video game development postmortems constitute an important source of empirical information regarding software engineering practices, production processes, organizational challenges, and project outcomes. These documents are typically produced by development teams after project completion and describe elements that contributed positively or negatively to the development process. Prior work by Politowski et al. [1] compiled a dataset of coded problem instances extracted from postmortems published between 1998 and 2019 and organized them within a structured taxonomy.

Since the publication of that dataset, additional postmortems have become available through Game Developer, Reddit, and GDC Vault. The present dataset was compiled to document these more recent materials while preserving compatibility with the original taxonomy and coding scheme.

The collection process combined source identification, taxonomy-based coding, AI-assisted candidate extraction, human validation, and expert review. This data article documents the procedures used to collect, code, validate, and integrate the data, and provides access to the resulting records, source materials, metadata, and validation documentation.

The dataset extends the temporal coverage of the original resource from 1998 to 2019 to 1998–2025 and provides accompanying documentation intended to facilitate reuse, replication, verification, and future dataset maintenance.

## 3 Data Description

The repository contains the complete dataset, source materials, validation records, and documentation used to construct the extended postmortem corpus. All files are provided through the Zenodo repository and are organized into separate folders corresponding to the major stages of data collection, validation, and integration. Table 1 summarizes the organization of the repository and the content of each folder.

**Table 1:** Repository structure and contents.

Folder	Description
Dataset	Merged dataset containing all coded problem instances from 1998 to 2025 in XLSX.
PostMortem	Original postmortem documents collected from Game Developer, Reddit, and GDC Vault in PDF and TXT formats.
Prompt	Prompt used for AI-assisted candidate problem extraction from postmortem documents.

The primary dataset is distributed in XLSX format. Each row corresponds to a single coded problem instance extracted from a game development postmortem. Table 2 summarizes the variables available in the released dataset. The released dataset contains 267 postmortems and 1227 coded problem instances. Of these, 927 instances originate from the baseline dataset published by Politowski et al., whereas 300 instances correspond to the 2019–2025 extension. Source provenance is preserved for all observations. Table 3 provides an overview of dataset composition. The repository also includes prompts used to extract data. These files are provided to facilitate navigation of the repository and traceability between source documents and coded observations.

**Table 2:** Variables included in the released dataset.

Variable	Description
id	Unique identifier of the coded problem instance.
SRC	Source dataset identifier.
title	Postmortem title.
year	Publication year.
source	Original source URL or reference.
name	Name of the video game.
platform	Target platform(s).
genre	Game genre.
mode	Gameplay mode.
group	High-level taxonomy category.
group4	Validated taxonomy category.
type	Original problem type classification.
type_refactored	Validated problem type classification.
quote	Supporting quotation.
File	Source document identifier.

**Table 3:** Dataset composition.

Source	Postmortems	Problem instances
Game Developer/Gamasutra	214	991
Reddit	25	99
GDC Vault	28	137
Total	267	1227

## 4 Experimental Design, Materials and Methods

This section presents the methodological framework used to extend and analyze the postmortem dataset while preserving comparability with prior work. We describe the data collection process, the integration of complementary sources to capture diverse development contexts, and the coding protocol applied uniformly across all instances. Attention is given to the balance between dataset expansion, methodological rigor, and potential sampling biases introduced by heterogeneous sources.

### 4.1 Sampling and coding protocol

We queried the same online venue as Politowski et al. to collect postmortems published between 2019 and 2025, removing duplicates as well as content that did not qualify as postmortems. Due to the limited number of additional postmortems available from the original source (14 postmortems), we complemented the dataset with 25 postmortems from Reddit and 28 additional postmortems from GDC Vault, yielding 300 new problem instances for the 2019–2025 period. All sources were processed using the same coding protocol to ensure consistency and comparability.

To preserve alignment with the baseline dataset of Politowski et al. [1], we strictly applied the original taxonomy without introducing new categories. This decision prevents category drift and ensures methodological consistency. Although the sources differ in editorial format, including professionally edited postmortems (Gamasutra/Game Developer), self-reported accounts (Reddit), and interview-based presentations (GDC Vault via YouTube),^1^ all selected content satisfies the definitional criteria of postmortems, namely the explicit discussion of “what went wrong and what went well”. We therefore followed the same selection criteria as Politowski et al. to identify eligible postmortems. Inclusion criteria were applied uniformly regardless of source, and all data were processed through an identical pipeline combining AI–assisted extraction with systematic human validation and expert consensus.

For Reddit and GDC Vault, potential postmortems were first identified using Copilot and subsequently filtered according to the same inclusion criteria. Only postmortems discussing “what went right and what went wrong” were retained.

From an initial set of 145 potential Reddit postmortem candidates, only 25 postmortems were retained. From an initial set of 50 GDC Vault candidates, 28 postmortems met the inclusion criteria and were retained.

All included postmortems were subjected to identical inclusion criteria and a uniform coding protocol, regardless of origin. Each document was filtered to ensure compliance with the postmortem definition, then processed through AI–assisted extraction followed by systematic human validation and expert consensus. This source-agnostic validation pipeline ensures that all retained problem instances meet the same standards of interpretive reliability. Nevertheless, we acknowledge that differences in narrative structure and level of detail between sources can influence the ease of problem identification, as reflected in the observed variation in AI–human agreement rates. Accordingly, source origin is treated as a potential confounding factor, and results are interpreted as aggregated trends rather than source-specific effects. The 2019–2025 extension contains a mixture of sources that differ in editorial format and development context. Consequently, comparisons with the baseline should be interpreted as descriptive comparisons between documented datasets rather than direct evidence of temporal change.

### 4.2 Definition of a problem instance

A problem instance was defined as a distinct issue explicitly described in a postmortem as having negatively affected the development process, the project, the team, or the resulting product. Each problem instance was associated with a supporting text span from the source document and assigned to one problem type within the taxonomy proposed by Politowski et al. When a single passage described multiple distinct problems, these were recorded as separate problem instances when they could be unambiguously mapped to different taxonomy categories.

### 4.3 Justification of complementary sources and associated biases

The inclusion of Reddit and GDC Vault as complementary sources is motivated by the need to capture heterogeneous development contexts that are underrepresented in traditional postmortem venues. Reddit primarily reflects experiences from independent developers and small studios, often characterized by limited resources, constrained visibility, and direct engagement with distribution platforms. In contrast, GDC Vault provides access to postmortems and talks originating from established studios, including AAA productions, where development processes are typically more structured and resource-intensive.

These contextual differences introduce potential sampling biases. Reddit-derived postmortems may overrepresent Business-related and Marketing challenges, reflecting the centrality of discoverability and monetization issues in independent development contexts. Conversely, GDC Vault content may underrepresent such concerns while emphasizing production scale coordination, technical integration, and pipeline complexity. As observed in the stratified analysis, the distribution of problem types varies significantly across sources, with Marketing issues being disproportionately represented in the Reddit subset.

These biases may affect the interpretation of aggregate results by amplifying source-specific problem profiles. To mitigate this limitation in future work, we recommend the use of source-aware weighting schemes, where observations are normalized according to source-specific distributions or rebalanced through stratified sampling. For instance, inverse-proportion weighting or equalized per-source sampling could be applied to ensure that no single development context disproportionately influences the overall distribution.

For each selected postmortem, the following coding protocol was applied:

AI-assisted coding: Microsoft 365 Copilot was employed as an initial screening tool to identify candidate problem instances for subsequent human validation.

Double human validation and consensus review following AI-assisted extraction.

Each candidate problem instance was first reviewed by the first author. The review assessed both the relevance of the identified problem and the consistency of its assigned taxonomy category with the supporting text span. Candidate instances that were unclear, incorrectly classified, or insufficiently supported by the source text were flagged for further review.

A second review was subsequently conducted with an experienced game-development practitioner with >20 years of professional experience. The reviewer examined all the proposed problem instances and their taxonomy assignments.

After this second evaluation, all disagreements between the initial coding and the expert assessment were discussed until consensus was reached.

The final dataset contains only problem instances for which independent review and subsequent consensus were achieved. Of the 300 AI-generated candidate instances, 265 (88.3 %) were validated without modification, whereas 35 (11.7 %) required reclassification following expert review. This validation process was designed to improve coding reliability while preserving taxonomy consistency across sources.

To quantify agreement between the initial Microsoft Copilot classification and the subsequent human validation, inter-rater reliability was assessed using Cohen's kappa coefficient *κ* [2]. Cohen's kappa was selected because it corrects for chance agreement and is widely used for categorical classification tasks. Agreement was calculated by comparing the initial AI-generated labels with the corresponding validated human labels prior to consensus review.

To provide an overview of the data collection and coding procedure, Fig. 1 summarizes the complete analysis pipeline. The process begins with the collection of postmortems from multiple sources, followed by AI-assisted extraction constrained by the original taxonomy. Each candidate problem instance is then subjected to systematic human validation and expert consensus to ensure interpretive reliability. The final validated dataset is subsequently used to compute aggregated distributions at both the Group and Type levels. In addition to supporting dataset extension, the AI-assisted step also provides an opportunity to evaluate the feasibility of using large language models for empirical dataset maintenance. The use of LLM-based tools can substantially accelerate the data extraction process. Recent empirical studies suggest that large language models can effectively support software engineering tasks through iterative human–AI collaboration, particularly in code generation and refactoring [3]. However, their limitations in instruction following and code correctness necessitate human oversight, especially in maintenance-intensive tasks such as dataset curation [4]. Hybrid approaches combining human expertise with LLM assistance have been shown to achieve performance comparable to human-only processes in annotation and data-related tasks [3].

(1) $$P_{e}=\sum_{i=1}^{k}p_{i}^{AI}p_{i}^{\mathrm{Human}}$$

**Fig. 1:**
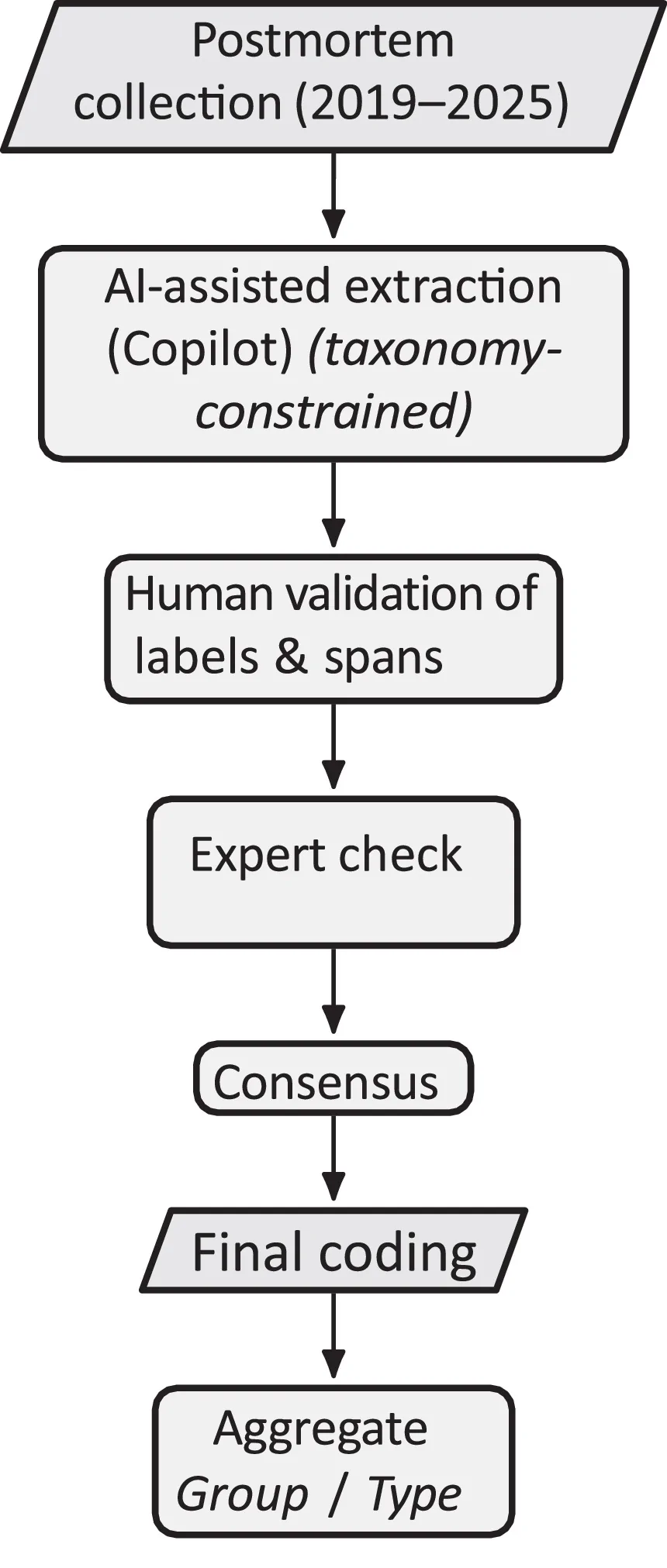
Coding protocol and analysis pipeline.

The expected agreement $\left(P_{e}\right)$, where $p_{i}^{AI}$and $p_{i}^{Human}$denote the marginal proportions associated with category i for Microsoft Copilot and the human validator, respectively, was calculated from the marginal distributions of the initial Microsoft Copilot classifications and the corresponding human-validated classifications across all 300 candidate problem instances. Agreement was computed from paired comparisons of the initial and validated classifications at both the Group and Problem Type levels prior to consensus review.

Following the standard formulation of Cohen's kappa, $P_{e}$corresponds to the sum of the products of the marginal proportions associated with each classification category, representing the probability of agreement expected by chance alone.

The observed agreement between the initial AI classifications and the human validation was $P_{o}$ = 0.883 (265 of 300 classifications). Inter-rater reliability between the initial Microsoft Copilot classification and the subsequent human validation was assessed using Cohen's kappa coefficient (κ), computed as:

(2) $$\kappa =\frac{P_{o}-P_{e}}{1-P_{e}}$$

The agreement expected by chance was $P_{e}$ = 0.2746. Substitution of these values into Eq. (2) yields:

$$\kappa =\frac{0.8833-0.2746}{1-0.2746}=0.839\approx 0.84$$

Cohen's kappa coefficient reached κ = 0.84, indicating a high level of agreement according to the benchmark proposed by Landis and Koch [2]. The high level of agreement observed between AI-assisted and human classifications supports the reliability and reproducibility of the proposed coding workflow.

During the coding process, we systematically reviewed each problem instance identified by the AI. These observations were later analyzed to assess the practical usefulness of AI in maintaining empirical software engineering datasets.

For the data from Gamasutra, corresponding to the expert consensus phase, most problem instances were confirmed. Only five out of 64 instances identified across 14 postmortems were reclassified based on direct quotations from the postmortems, observed agreement was 92 %.

For the data from Reddit, the same analysis process was applied to the 25 retained postmortems. A substantial proportion of problem instances were confirmed (77 out of 99), while 22 out of 99 instances were reclassified during the human validation process based on direct quotations from the posts. Observed agreement was 77.8 %.

For the data from GDC Vault, the same analysis process was applied to the 28 retained postmortems. A proportion of problem instances were confirmed (129 out of 137), while eight out of 137 instances were reclassified during the human validation process based on direct quotations from the posts. This corresponds to an observed agreement of 94.2 %. The level of agreement can be attributed to the discussion format provided by the GDC Vault structure.

Across the three data sources, the AI-assisted coding workflow produced 300 candidate problem instances. Following systematic human validation and expert consensus, all 300 instances were retained in the final dataset, indicating that the AI successfully identified relevant problem statements for every candidate record. Nevertheless, 35 instances (11.7 %) required reclassification to a different taxonomy category following expert review, while 265 instances (88.3 %) were confirmed without modification.

Most reclassifications reflected ambiguities between conceptually adjacent taxonomy categories rather than failures in problem identification. The validation process therefore served to refine taxonomy assignments and improve classification consistency while preserving the complete set of identified problem instances.

Problem instances were extracted using Microsoft 365 Copilot (accessed May 2026). Because Microsoft does not disclose the exact underlying model version, configuration, or inference parameters, these details cannot be reported. Reproducibility was supported by applying a fixed prompt protocol throughout the extraction process. The exact prompt used during candidate extraction is provided in the file prompt.docx available in the Zenodo repository.

The same prompt was used to generate all candidate problem instances, which were subsequently reviewed according to the validation procedure described above.

To ensure traceability of the dataset and the construction process, Table 4 summarizes the number of postmortems, AI-generated candidate instances, human-confirmed instances, reclassified instances, and final validated instances for each source. This reconciliation provides an explicit accounting of all problem instances included in the 2019–2025 extension and distinguishes the newly collected data from the baseline dataset of Politowski et al. The source-level counts sum to 300 problem instances, of which 265 were confirmed without reclassification and 35 required reclassifications during human validation.

**Table 4:** Dataset reconciliation and validation summary.

Source	Postmortems	Candidates	Confirmed	Reclassified	Final
Gamasutra/Game Developer	14	64	59	5	64
Reddit	25	99	77	22	99
GDC Vault	28	137	129	8	137
Extension (2019–2025)	67	300	265	35	300
Baseline (1998–2019)	200	–	–	–	927
Merged dataset	267	–	–	–	1227

### 4.4 Data records

The released dataset is provided as a structured spreadsheet in which each row corresponds to a single coded problem instance extracted from a game development postmortem. Each record contains both descriptive metadata about the game and the source document, as well as the taxonomy labels assigned during the coding process. The dataset preserves the original problem taxonomy proposed by Politowski et al. while extending coverage to postmortems published between 2019 and 2025. For each problem instance, the dataset stores the original quotation supporting the classification, enabling traceability and independent verification of coding decisions. Table 2 summarizes the variables included in the released dataset.

To facilitate dataset exploration and reuse, Table 5 summarizes the main descriptive characteristics of the released dataset, including corpus size, source provenance, temporal coverage, and the principal metadata dimensions available for each observation. This overview provides additional context regarding the composition and coverage of the dataset.

**Table 5:** Descriptive characteristics of the released dataset.

Variable	Category	Count
Dataset size	Postmortems	267
	Problem instances	1227
Source	Game Developer / Gamasutra	214
	Reddit	25
	GDC Vault	28
Publication period	1998–2019 (baseline dataset)	200 postmortems
	2019–2025 (extension dataset)	67 postmortems
Dataset type	Baseline problem instances	927
	Extension problem instances	300
Variables available	Year	Included
	Platform	Included
	Genre	Included
	Gameplay mode	Included
	Source provenance	Included
	Supporting quotation	Included

To provide a more detailed overview of the corpus, Table 6 summarizes the distribution of records across the main descriptive dimensions available in the dataset, including platform, genre, and gameplay mode. Because some games were released on multiple platforms and support more than one gameplay mode, percentages should be interpreted as proportions of coded records rather than mutually exclusive categories.

**Table 6:** Distribution of records across platform, genre, and gameplay mode.

Variable	Category	Count	%
Platform			
	PC	843	52.4
	Console	512	31.8
	Mobile	250	15.5
Genre			
	Action	321	20.0
	Strategy	177	11.0
	Adventure	174	10.8
	Shooter	152	9.5
	Simulation	140	8.7
	Platformer	120	7.5
	RPG	118	7.3
	Puzzle	77	4.8
	Roguelike	54	3.4
	Racing	50	3.1
	Sports	28	1.7
Gameplay mode			
	Single-player	1039	69.4
	Multiplayer	365	24.4
	Online	93	6.2

### 4.5 Distributions by problem type

Fig. 2 presents the original distribution of problems by Problem type, as proposed by Politowski et al., while Fig. 3 depicts the distribution observed between 2019 and 2025. No additional categories were introduced when analyzing problem types for the new period.

**Fig. 2:**
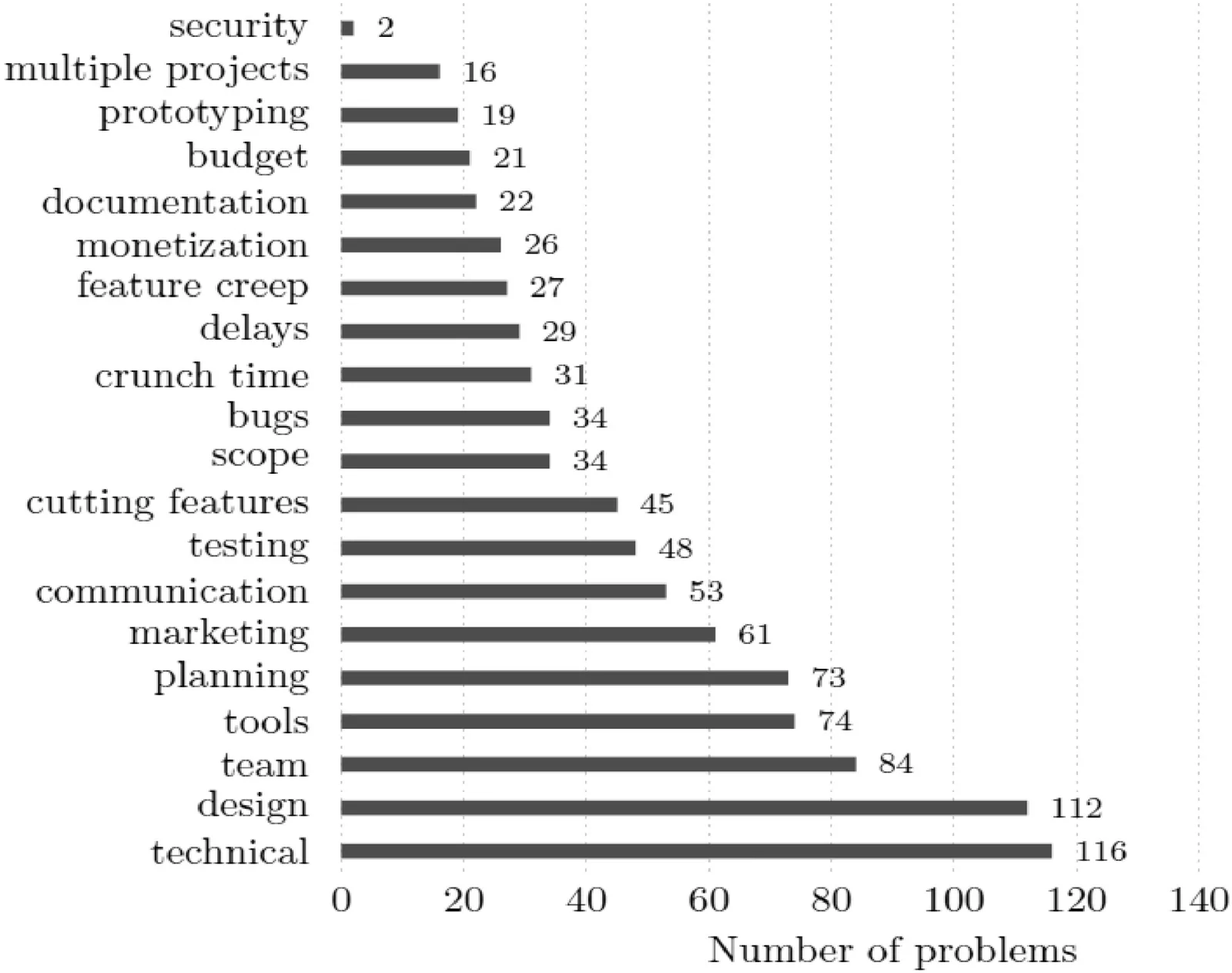
Distribution of problem types 1998–2019 baseline dataset.

**Fig. 3:**
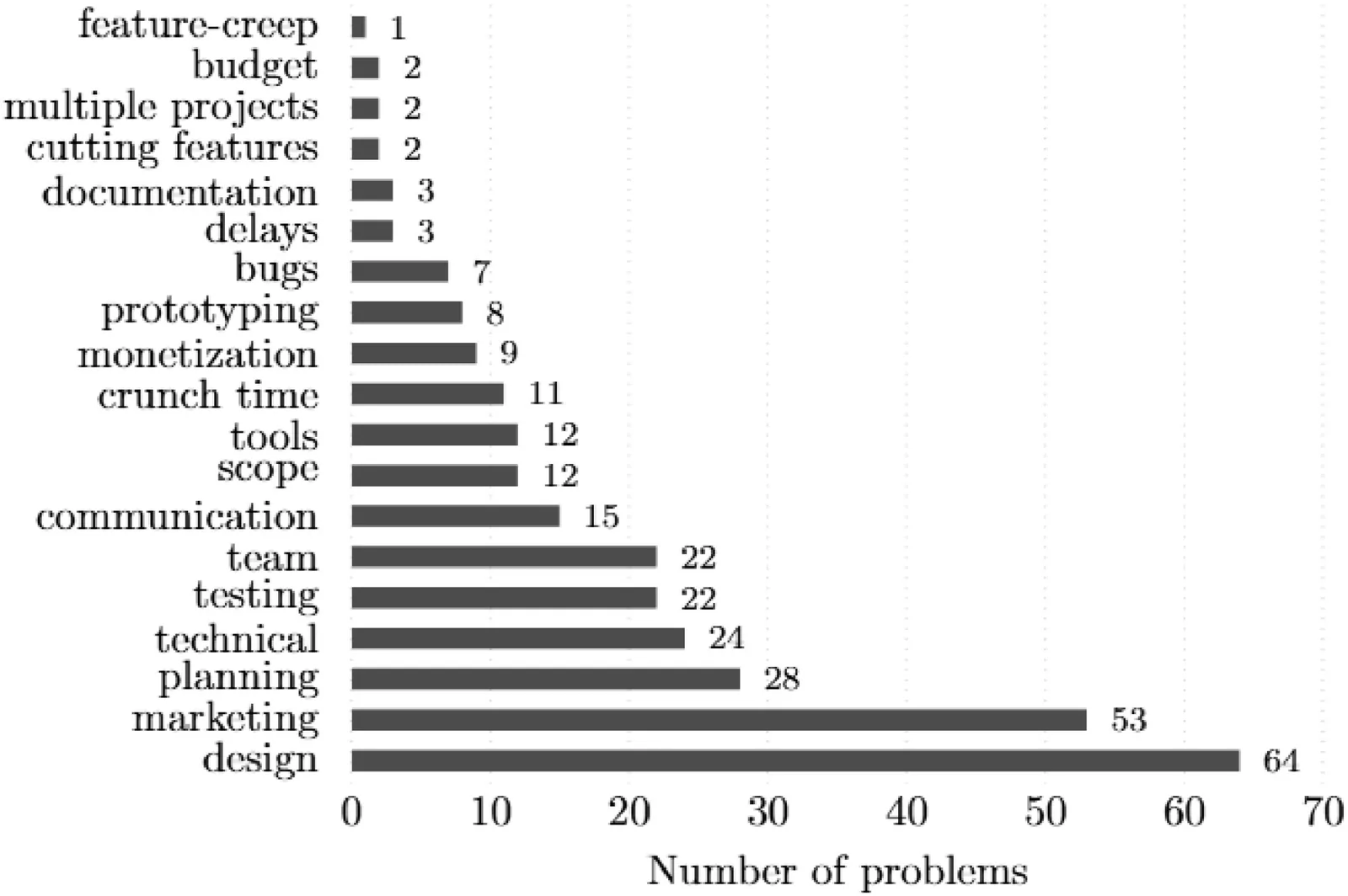
Distribution of problem types 2019–2025.

### 4.6 Dataset composition by source

To assess whether distributional shifts occurred, we conducted a post-hoc stratified analysis comparing problem distributions separately for the three constituent subsets: the Gamasutra/Game Developer subsample (n = 64 problem instances, 14 postmortems), the Reddit subsample (n = 99 problem instances, 25 postmortems), and the GDC Vault subsample (n = 137 problem instances, 28 postmortems).

At the Problem type level, the most critical divergence concerns Marketing, which represents 12.5 % of Gamasutra instances but 43.4 % of Reddit and 5.1 % of GDC Vault instances, a difference of 30.9 percentage points. The overall Marketing increase observed in the 2019–2025 extension is driven primarily by the Reddit subsample and should not be interpreted as a uniform industry-wide trend.

These stratified results also inform the temporal coverage. The Gamasutra subsample spans 2020–2024 (no postmortems were identified for 2019 or 2025), whereas the Reddit subsample provides broader year coverage from 2019 to 2025, including 12 instances from 2025 absent from the Gamasutra stratum.

Taken together, the stratified analysis reveals that the three sources contribute structurally different problem profiles. Per-stratum counts remain too limited for inferential comparison; each cell would average fewer than five observations under a full 17-category by three-sources contingency table, but the directional patterns are sufficiently consistent to treat source origin as a substantive moderating variable rather than a mere editorial confound.

## Limitations

The baseline dataset of Politowski et al. was recovered by downloading the replication package associated with the original publication [1], which includes a structured spreadsheet of all coded problem instances. Each record was verified against the original publication to confirm category labels and postmortem identifiers. Small divergences (<7.3 %) were detected between the downloaded replication package and the counts reported in the original article.

The replication package associated with Politowski et al. contained 1000 coded records, whereas the original publication reports 927 validated problem instances. Manual inspection indicated that this discrepancy originates from records present in the archived dataset that were not included in the final analytical counts reported in the publication. Because the original article consistently reports 927 validated problem instances, we adopted this value as the reference baseline for all comparative analyses presented in this paper. The additional records were preserved in the archived materials released with this study for transparency purposes but were excluded from the analytical baseline to maintain full comparability with the published results of Politowski et al.

The reliability coefficient was computed between the initial AI-generated classification and the validated human classification. As the coding protocol relied on AI-assisted preclassification rather than fully independent human coders, the reported kappa should be interpreted as a measure of agreement within a human-in-the-loop annotation workflow.

The validated baseline of 927 problem instances was merged with the 2019–2025 extension containing 300 validated problem instances, resulting in a merged analytical dataset of 1227 problem instances covering the period 1998–2025.

External validity. We acknowledge that the three sources exhibit different levels of structural regularity, as reflected in the divergent AI–human agreement rates observed during validation (92 % for Gamasutra, 77.8 % for Reddit, and 94 % for GDC Vault). This difference may be associated with the greater narrative consistency of professionally edited postmortems rather than with a systematic difference in the types of problems reported. Critically, all coding decisions were finalized through two rounds of human validation and expert consensus, which serves as a source-agnostic quality control mechanism that mitigates the effect of editorial heterogeneity on label reliability. A post-hoc stratified analysis confirms that the three sources exhibit structurally different problem profiles: per-stratum counts remain insufficient for inferential comparison at the Problem type level, but directional patterns are consistent enough to treat source origin as a substantive moderating variable. Aggregation in the primary analysis therefore represents a deliberate trade-off between statistical power and source homogeneity, acknowledged here as a residual threat to external validity pending replication on a source-balanced corpus.

## Ethics Statement

This article complies with Elsevier’s ethical guidelines and GDPR/CCPA regulations. All source materials were obtained from publicly accessible resources and were used in accordance with the access conditions established by the originating platforms. The dataset does not redistribute copyrighted source content; it contains only derived metadata, annotations, and references to original sources. Source-specific rights and usage conditions are documented for each category of collected material:

• Game Developer (Gamasutra): Professionally edited articles published with studio approval.
• Reddit: Participant-level personal information was not collected, stored, or redistributed. Only publicly available content was used, and all quotations are attributed to their original publicly accessible sources. Reddit usernames were removed from all released materials. No personal identifiers are included in the repository. All quoted material complies with Reddit’s public content and redistribution policies.
• GDC Vault: Conference talks from established studios (publicly accessible).
• No sensitive or personal data were collected. The dataset contains:
• Aggregated problem instances (267 postmortems, 1227 instances)
• Anonymized metadata (year, source, problem type)
• Publicly available quotations (with source attribution)
• Public: Dataset released under CC BY 4.0.
• Reviewers: Full access via DOI:10.5281/zenodo.19683430

## Credit Author Statement

**Claude Cardinal**: Conceptualization, Methodology, Software, Validation, Formal analysis, Investigation, Data curation, Writing Original Draft, Writing – Review & Editing, Visualization. **Bruno Bouchard**: Conceptualization, Methodology, Validation, Resources, Writing – Review & Editing, Supervision, Project administration.

## Footnotes

1 Official sources: Game Developer (formerly Gamasutra): https://www.gamedeveloper.com; Reddit: https://www.reddit.com; GDC Vault: https://gdcvault.com.Official sources: Game Developer (formerly Gamasutra): https://www.gamedeveloper.com; Reddit: https://www.reddit.com; GDC Vault: https://gdcvault.com.
